# Exploring social determinants of health: Comparing lower and higher income individuals participating in telepsychiatric care for depression

**DOI:** 10.3389/fpsyt.2022.1026361

**Published:** 2023-01-05

**Authors:** Heather G. Belanger, Mirène Winsberg

**Affiliations:** ^1^Brightside Health Inc., Oakland, CA, United States; ^2^Department of Psychiatry and Behavioral Neurosciences, University of South Florida, Tampa, FL, United States

**Keywords:** social determinants of health (SDOH), depression, antidepressant, low income, SDOH, telepsychiatry, outcome, United States

## Abstract

**Background:**

Telemental health may increase access to care; there has been little research on efficacy with those at the lower end of the income distribution. The purpose of this study was to determine whether lower vs. higher income patients receiving telepsychiatric care for depression achieve: (1) effective symptom reduction and (2) similar outcomes.

**Methods:**

Data utilized were obtained from a national mental health telehealth company and consisted of 5,426 U.S.-based patients receiving psychiatric care for moderate to severe depression between October, 2018 and January, 2022. Propensity matching was used to create lower and higher income samples (*n* = 379 in each) using 22 covariates. These samples were then compared using repeated measures ANOVA on Patient Health Questionnaire-9 (PHQ-9) scores at start of treatment, 6, 8, 10, 12, 14, and 16 weeks.

**Results:**

Both lower and higher income groups made significant improvement over time, with groups averaging mild symptom severity by week 16. There was a significant group x time interaction, such that the lower income group had significantly greater depression severity at the last two timepoints.

**Conclusion:**

Lower and higher income groups both made significant improvement in depression symptom severity over time following initiation of psychiatric treatment via a telehealth platform, though higher income individuals, all else being equal besides employment, tend to do better. These findings suggest that when lower income individuals do participate in care, good outcomes can be achieved. Further research is needed to better understand the role social determinants of health (SDOH) play in outcome disparities.

## Introduction

Major depressive disorder (MDD) is one of the most prevalent (1) and consequential health disorders in the country. It is one of the leading causes of disability in the United States, though only about 65% of people with depression receive treatment (2). Due to fewer resources and access, barriers to care may be even greater in lower income patients. It is conceivable that those with lower incomes may lack digital literacy or high-quality internet connection and may further have lower awareness of telepsychiatry.

In general, lower income individuals have worse health and mental health outcomes (3–6). Some have posited this is due to lower medication adherence, greater mistrust of healthcare providers, and lower quality of care (7, 8). While evidence is mounting that digital mental health care options are effective and can help eliminate structural barriers to evidence-based care, (9–19) there is a consensus that more research is needed with respect to providing telemental health services to lower income patients (20).

The goal of the current study is to determine whether lower income patients being treated for depression via a telemental health platform achieve: (1) effective symptom reduction and (2) similar outcomes as higher income patients.

## Materials and methods

### Participants

Participant data utilized in the current investigation were obtained from a national mental health telehealth company (i.e., Brightside) and consisted of 5,426 U.S.-based adult patients, aged 18 to 80 (mean age = 33.67, sd = 9.72) receiving psychiatric care for depression between October, 2018 and January, 2022. These data were a subset from another study (21) examining the impact of age. Participants were eligible if they (a) were diagnosed with Major Depressive Disorder by their provider (b) had moderate to severe symptom severity at intake (PHQ-9 ≥ 10) (c) had complete income data, (d) were prescribed at least one psychiatric medication (described below), and (e) had complete outcome data. Patients at high risk for suicide, and patients with psychosis or in need of emergency psychiatric services at the initial evaluation were not eligible.

### Procedure

All study procedures were approved by the WCG Institutional Review Board for the retrospective analysis of patient data obtained by Brightside as part of routine clinical care. Enrolled Brightside patients complete an initial digital intake that includes clinically validated measures of depression and anxiety, as well as questions about clinical presentation, medical history, and demographics. All Brightside patients are required to complete baseline and intake questionnaires. During a patient’s first session, a licensed professional prescribed psychiatric medication(s) for each patient. Over the course of treatment, patients communicated with their provider both asynchronously via messaging and synchronously via video telehealth sessions. Brightside also uses a measurement-based approach to tracking long-term outcomes by prompting patients to complete periodic assessments during treatment. Assessments were completed at baseline/intake, and periodically thereafter. Surveys were administered digitally through an email prompt. Survey completion at baseline, 6 weeks, 8 weeks, 10 weeks, 12 weeks, 14 weeks, and 16 weeks were required for participation.

### Measures

The Patient Health Questionnaire-9 (PHQ-9) is a 9-item self-report measure used to assess the severity of depressive symptoms present within the prior 2-weeks as outlined by DSM-5 criteria. Respondents rate items on a 4-point Likert scale (0-3) and total scores range from 0 to 27, with >9 indicating mild-to-low symptoms and 10 + indicating moderate-to-severe symptoms (22). Sample items include: “Little interest or pleasure in doing things,” and “Feeling down, depressed, or hopeless.” The PHQ-9 shows strong reliability, demonstrating 88% sensitivity and 88% specificity for Major Depressive Disorder (22). There is also evidence that the PHQ-9 can be used as a measure of antidepressant response (23). It has demonstrated reliability and validity across various cultures and settings (24–26). PHQ-9 scores were collected via self-report electronically at baseline, and at weeks 6, 8, 10, 12, 14 and 16 and served as the outcome measure of interest. As part of the PHQ-9, patients were asked to what extent, if they scored >0, these problems have made it difficult for them in four areas – social, family, work, and activities, on a scale from 0 to 3, with 0 indicated “not difficult at all,” 1 – “somewhat difficult,” 2 – “very difficult,” and 3 – “extremely difficult” (27). These were summed to create a measure of the functional impact of depression (27).

The GAD-7 is a 7-item self-report measure of Generalized Anxiety Disorder (GAD) symptoms with a four-point Likert scale and a total score ranging from 0 to 21. Sample items include: “Feeling nervous, anxious or on edge,” and “Trouble relaxing.” Like the PHQ-9, a higher score corresponds to a greater anxiety severity. The GAD-7 has good psychometric properties with 89% sensitivity and 82% specificity for GAD (28, 29). It was included in this study to equate groups on anxiety severity.

Other standard demographic, health, and clinical information was also collected at baseline, such as age, sex, education, race/ethnicity, employment status, census-based geographic region of the country, prior episodes of depression (none, one, or more than one), duration of the current episode, any prior mental health treatment (yes/no), primary non-mood symptom complaint (agitation, concentration, motivation, sleep, none), frequency of social media use from 0 to 4 (i.e., never, rarely, several times/week, once/day, several times/day), current participation in concurrent psychotherapy, frequency of technology use on a scale from 0 to 4 for personal (non-work) use (e.g., phone, tablet, computer, gaming console), and total number of chronic health conditions endorsed (including arrhythmia, asthma, cancer. hypercholesterolemia, diabetes, heart condition, irritable bowel syndrome or Crohn’s disease, lung disease, obesity, thyroid disease, eating disorder, and chronic pain/fibromyalgia).

### Interventions

Because this is a naturalistic sample, participants were prescribed a variety of medications. The most commonly prescribed medication category of the sample (61.5%) was selective serotonin reuptake inhibitors (SSRIs), followed by norepinephrine and dopamine reuptake inhibitors (NDRIs, 20.7%), serotonin-norepinephrine reuptake inhibitor (SNRI, 5.5%), trazodone (or trazodone + SSRI) (4.9%), SSRI and NDRI combination (4.4%), mirtazapine (or mirtazapine + SSRI) (1.5%), and atypical antipsychotics and SSRI combination (1.5%). The dosage of index antidepressants remained relatively consistent throughout the study period and were prescribed in standard therapeutic ranges. Dosage adjustments were made based on participant responses to the PHQ-9 and other assessments, as well as virtual visits between participation and providers. Because specifics about treatment were not the focus of this study and because this was a naturalistic study, medications and dosages were not controlled and therefore varied to meet individual needs. 26% of the sample was concurrently engaged in psychotherapy.

### Data analyses

Data analyses were performed via SPSS, Version 28. Two income-defined groups were created, one group with annual incomes below $30,000 and one group with annual incomes above $60,000. Comparisons between groups were made using t-tests for continuous variables and chi-square analyses for categorical and evaluated at *p* < 0.01. Propensity-matching of the two groups using 0.0009 caliper was done based on *a priori* variables collected at baseline that might potentially affect outcome (30). This approach attempts to replicate a randomized trial by obtaining treatment groups with similar distributions of known covariates (31). Included variables were: age, sex, race/ethnicity, education level, employment status, census-defined region of the country, primary non-mood symptom complaint (agitation, concentration, motivation, sleep, none), past/present use of antidepressant medication, history of any prior mental health treatment, total number of chronic medical conditions (arrhythmia, asthma, cancer, hypercholesterolemia, chronic pain, diabetes, fibromyalgia, heart condition, irritable bowel syndrome/Crohn’s disease, lung disease, thyroid disease, obesity), current smoker, prior depression (yes/no), duration of depression, baseline depression and anxiety symptom severity, functional impact of depression rating at baseline, frequency of social media use from 0 to 4 (i.e., never, rarely, several times/week, once/day, several times/day), current participation in concurrent psychotherapy, and frequency of technology use on a scale from 0 to 4 for personal (non-work) use (e.g., phone, tablet, computer, gaming console). Repeated measures analysis of variance (ANOVA) was used to compare the groups over time (at baseline, and at weeks 6, 8, 10, 12, 14, and 16) on total PHQ-9 scores over time. Mauchly’s test was used to test the sphericity assumption, with the Greenhouse–Geisser correction (32) used for violations.

## Results

In the entire sample, there were 3,186 individuals in the higher income group and 2,240 in the lower income group. Besides income, these groups differed significantly on several variables. The lower income group had significantly more severe depressive symptoms at baseline, *t* = 14.40, more severe anxiety symptoms at baseline, *t* = 7.69, greater reported functional impact of depressive symptoms, *t* = 7.26, fewer average number of chronic medical conditions, *t* = 4.51, and was significantly younger than the higher income group, *t* = 35.90, all *p* < 0.001. The lower income group also had a greater proportion who were female, χ^2^ = 30.29, *p* < 0.001, less degree of education/number of graduate degrees, χ^2^ = 1112.68, *p* < 0.001, greater number of minorities, χ^2^ = 73.67, *p* < 0.001, more people who were unemployed, χ^2^ = 1642.97, *p* < 0.001, fewer who had had one prior depressive episode, χ^2^ = 43.61, *p* < 0.001, fewer who had had prior mental health treatment, χ^2^ = 13.43, *p* < 0.001, longer duration of depression, χ^2^ = 117.29, *p* < 0.001, more people endorsing motivation/low energy, χ^2^ = 10.02, *p* = 0.002, fewer people endorsing agitation, χ^2^ = 33.61, *p* < 0.001, more who endorsed smoking, χ^2^ = 67.58, *p* < 0.001, fewer people currently receiving psychotherapy, χ^2^ = 82.50, *p* < 0.001, more people endorsing technology use multiple times per day, χ^2^ = 173.50, *p* < 0.001, and more people endorsing social media use once per day, χ^2^ = 34.07, *p* < 0.001. The two income groups did not significantly differ on region of the country, or endorsement of sleeping or concentration difficulties. Please see Table 1 for a summary of the initial sample.

**TABLE 1:** Characteristics of lower and higher, entire sample (*N* = 5,426).

Characteristic	Lower income	Higher income	*t* or χ^2^	Effect size^a^	*P*-value
Age	28.59 (8.25)	37.24 (9.06)	35.90	8.73	<0.001
**Sex**			30.29	0.08	<0.001
Male	27%	35%			
Female	73%	65%			
**Education:**			1,112.68	0.45	<0.001
No high school	3%	0.1%			
High school diploma	49%	14%			
Some college	14%	11%			
College degree	27%	44%			
Graduate degree	7%	31%			
**Race/Ethnicity**			73.67	0.12	<0.001
White/Caucasian	73%	81%			
Asian	4%	4%			
Hispanic	11%	6%			
Black/African American	5%	5%			
Other	7%	4%			
**Employed**			1,642.97	0.55	0.000
Full time	31%	85%			
Part time	26%	3%			
Unemployed	43%	12%			
**Region of the country**			8.84	0.04	0.03
Midwest	15%	13%			
Northeast	17%	20%			
South	40%	39%			
West	28%	28%			
**Prior episodes of depression**			43.61	0.09	<0.001
None	40%	37%			
One	9%	14%			
More than one	51%	49%			
Prior mental health treatment	25%	29%	13.43	0.05	<0.001
Number of chronic medical conditions	0.53 (0.85)	0.64 (0.89)	4.51	0.87	<0.001
Baseline PHQ-9	19.11 (4.24)	17.43 (4.22)	14.40	4.23	<0.001
Baseline GAD-7	15.14 (4.56)	14.14 (4.80)	7.69	4.70	<0.001
Functional impact total	9.95 (1.93)	9.54 (2.08)	7.26	2.02	<0.001
**How long depressed**			117.29	0.15	<0.001
Less than 2 weeks	<1	%1%			
2 weeks to 2 months	10%	13%			
2 months to 1 year	24%	32%			
1 to 2 years	16%	18%			
More than 2 years	50%	36%			
**Primary non-mood symptom**					
Sleep	5%	5%	0.02	0.00	0.88
Motivation/Low Energy	37%	32%	10.02	0.04	0.002
Agitation/Irritability	9%	15%	33.61	0.08	<0.001
Concentration	5%	7%	5.53	0.03	0.02
None	<1%	<1%	0.16	0.01	0.69
Current smoker	15%	8%	67.58	0.11	<0.001
**Current treatment**			82.50	0.12	<0.001
Medication	80%	70%			
Medication + Therapy	20%	30%			
**Frequency of technology use, 0-4**			173.50	0.18	<0.001
Seldom, Never	2%	3%			
Rarely	5%	11%			
Few times/Week	13%	21%			
Once/Day	17%	19%			
Multiple times/Day	63%	46%			
**Social media use, 0-4**			34.07	0.08	<0.001
Seldom, Never	8%	9%			
Rarely	14%	18%			
Few times/Week	10%	11%			
Once/Day	63%	55%			
Multiple times/Day	5%	7%			

^a^Effect sizes are Cohen’s d for continuous variables and Cramer’s V for categorical variables. Effect sizes are interpreted as small (0.2), medium (0.5), and large (0.8) (38).

Mean values are presented for continuous variables (with standard deviations in parentheses) and frequency counts are presented (with%) for categorical variables.

**FIGURE 1:**
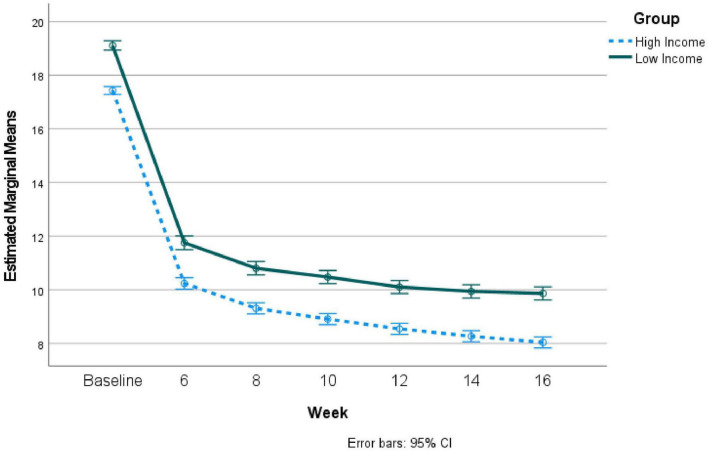
Repeated measures results comparing depression severity over time during telepsychiatry treatment for high vs. low income groups: Non-matched groups.

A repeated measures analysis of variance (ANOVA) comparing the lower and higher income groups on depression severity across time revealed that PHQ-9 scores differed significantly across time, *F* = 4913.54, *p* < 0.000, η^2^ = 0.48, such that scores significantly decreased over time. There was no significant group x time interaction, *F* = 1.53, *p* = 0.17, η^2^ = 0.000, though the low income group reported significantly greater depressive symptom severity overall, *F* = 149.69, *p* < 0.001, η^2^ = 0.03. Please see Figure 1 for an illustration of these results.

**FIGURE 2:**
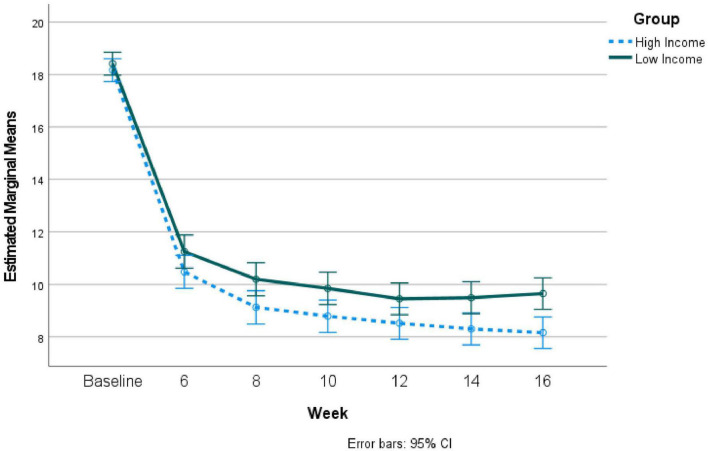
Repeated measures results comparing depression severity over time during telepsychiatry treatment for high vs. low income groups: Propensity-matched groups.

Due to the many differences between groups at baseline, propensity matching was used to create matched groups with 379 in each group. Despite matching, the groups still significantly differed on employment, χ^2^ = 59.83, *p* < 0.001, such that the lower income group had fewer fully employed individuals. There were no other differences between the groups on assessed variables. Repeated measures ANOVA comparing the lower and higher income groups on depression severity across time revealed that PHQ-9 scores differed significantly across time, *F* = 696.88, *p* < 0.001, η^2^ = 0.480, such that scores significantly decreased over time. There was a significant group x time interaction, *F* = 7.43, *p* < 0.007, η^2^ = 0.01, such that the lower income group had significantly greater depression severity over time at the last two timepoints. Please see Figure 2 for these results. As can be seen in Figure 2, both groups had PHQ-9 scores less than 10 by week 10 and beyond.

## Discussion

This study demonstrates that individuals with both lower income (i.e., below $30,000 per year) and higher income (i.e., above $60,000 per year) receiving completely virtual treatment for major depression achieve significant symptom reduction across 16 weeks, going from a moderate to severe level of symptom severity to a level considered mild (22). While there may be an assumption that lower income individuals will not benefit from telepsychiatry services due to poorer access, this assumption must be tested by distinguishing between access and outcome. Contrary to this assumption, in the current sample of individuals being treated for depression by a national mental health telehealth company, the lower income group reported using technology (e.g., phone, tablet, computer, and gaming console) more than the higher income group. It is unclear why that may be the case, but the greater rate of unemployment among the lower income group may provide them with more time. Notably this is a group of individuals who elected to pursue telemental health platform for their care, so this particular group of lower income individuals were obviously able to access it, which may not be the case for some (33). However, a recent study, in accordance with this sample, found that people with lower incomes (less than $25,000 annually) were more likely to use telehealth services during the pandemic than people with higher incomes (34) suggesting that telehealth increases access for lower income individuals. Given that this study was conducted during the pandemic, the results may have altered the proportions of low vs. high income individuals accessing these services, though it is impossible to know with certainty.

Despite both groups showing significant improvement over time, the higher income group showed significantly greater improvement in the latter time periods, relative to the lower income group. There is very little research on efficacy of telemental health with lower income individuals. Studies of depression treatments conducted with lower income, homebound older adults, have demonstrated efficacy in reducing both depression and disability among disabled older adults (35–37), though these studies did not compare lower with higher income individuals. Further research is needed to better understand the role social determinants of health (SDOH) play in outcome disparities. For example, medication adherence was not addressed by this study and may be explanatory, as just one possibility. A prior study (21) using some of the same participants suggested no difference in outcome based on age. This study, in contrast, suggests that unlike age, socioeconomic status may affect outcome. A trial with only low-income individuals, randomized to different levels of intensity, therapeutic approach, etc., might elucidate ways to improve outcomes for lower income individuals.

Limitations of this study include selection bias, such that results may not apply to all adults. Conceivably those who opt into treatment by a telemental health provider are inherently more comfortable with technology and may therefore be in a better position to benefit from it. In addition, this study lacked a control condition not receiving care, preventing any comparative conclusions regarding the effect of treatment. Additionally, the number of asynchronous and synchronous messages was not controlled which could have impacted results.

In conclusion, lower and higher income groups both made significant improvement in depression symptom severity over time following initiation of psychiatric treatment via a telehealth platform, though higher income individuals, all else being equal besides employment, tend to do better. Further research is needed to better understand the role social determinants of health (SDOH) play in outcome disparities, as well as how best to increase access and engagement among lower income individuals.

## Data availability statement

The original contributions presented in this study are included in the article/supplementary material, further inquiries can be directed to the corresponding author.

## Ethics statement

The study involving humans were reviewd and approved by the WCG Institutional Review Board for the retrospective analysis of patient data obtained by Brightside as part of routine clinical care. The ethics committee waived the requirement of written informed consent for participation.

## Author contributions

HB contributed to the manuscript development, writing, and analyses. MW contributed to the manuscript development and writing. Both authors contributed to the article and approved the submitted version.
